# Contribution of Dendritic Cells in Protective Immunity against Respiratory Syncytial Virus Infection

**DOI:** 10.3390/v12010102

**Published:** 2020-01-15

**Authors:** Hi Eun Jung, Tae Hoon Kim, Heung Kyu Lee

**Affiliations:** 1 Graduate School of Medical Science and Engineering, Korea Advanced Institute of Science and Technology (KAIST), Daejeon 34141, Korea; euphoric@kaist.ac.kr; 2 Department of Internal Medicine, Gyeongsang National University Changwon Hospital, Changwon 51472, Korea; plm.dr.th.kim@gmail.com; 3 KAIST Institute for Health Science and Technology, KAIST, Daejeon 34141, Korea

**Keywords:** dendritic cells, respiratory syncytial virus, immunomodulation

## Abstract

Respiratory syncytial virus (RSV) is a major cause of severe respiratory disease in infants and the elderly. The socioeconomic burden of RSV infection is substantial because it leads to serious respiratory problems, subsequent hospitalization, and mortality. Despite its clinical significance, a safe and effective vaccine is not yet available to prevent RSV infection. Upon RSV infection, lung dendritic cells (DCs) detecting pathogens migrate to the lymph nodes and activate the adaptive immune response. Therefore, RSV has evolved various immunomodulatory strategies to inhibit DC function. Due to the capacity of RSV to modulate defense mechanisms in hosts, RSV infection results in inappropriate activation of immune responses resulting in immunopathology and frequent reinfection throughout life. This review discusses how DCs recognize invading RSV and induce adaptive immune responses, as well as the regulatory mechanisms mediated by RSV to disrupt DC functions and ultimately avoid host defenses.

## 1. Introduction

Respiratory syncytial virus (RSV) is an enveloped, single-stranded RNA virus that belongs to the Pneumoviridae family [1]. RSV was first isolated from chimpanzee with coryza and named Chimpanzee Coryza Agent (CCA) in 1956 [2]. In 1957, Chanock and colleagues recovered two viruses from infants with severe lower respiratory illness and reported that these viruses were indistinguishable from the CCA virus [3,4]. Thus, CCA was renamed RSV, and has been reported as the major cause of respiratory illness and morbidity in infants and children. RSV infection can lead to serious respiratory problems in vulnerable populations, such as children younger than one year of age and immunocompromised older adults. Furthermore, hospitalization and mortality associated with RSV impose economic and humanistic burdens on society [5,6].

While most acute respiratory viral infections, such as influenza, elicit long-term durable immune responses, RSV infection only leads to relatively short-lived protective immunity, which is why frequent RSV reinfection can occur throughout a patient’s life [7]. Several attempts have been made to develop an effective RSV vaccine. However, no vaccine exists today because candidates failed to induce persistent immune responses against RSV antigen without causing vaccine-associated disease enhancement [8]. 

Dendritic cells (DCs), which participate in innate immunity, are professional antigen-presenting cells that play an essential role in activating adaptive immune responses. During viral infection, DCs detect viruses via the innate receptors discussed below and process the viral antigens into peptides, which are presented to T cells in a complex with MHC molecules as epitopes. In addition, DCs affect the generation of a protective antibody response by fine-tuning CD4^+^ T cell polarization [9,10,11,12].

Lung DCs are classified into conventional DC1 (cDC1), cDC2, and plasmacytoid DC (pDC) [13]. cDC1s are generally considered as the primary subset that cross-presents antigens to CD8^+^ T cells, while cDC2s mediate CD4^+^ T cell priming, and pDCs are well known as producers of type I interferon (type I IFN) [14]. Each DC subset is widely distributed throughout the lungs. cDCs migrate to lung-draining lymph nodes when they recognize invading pathogens to initiate protective immune responses. In this review, we discuss how DCs recognize RSV infection and mediate anti-RSV immune responses, as well as the immunomodulatory strategies that RSV utilizes to avoid host defense mechanisms via DC regulation.

## 2. Virological Features of RSV and the Immune Response

**Figure 1:**
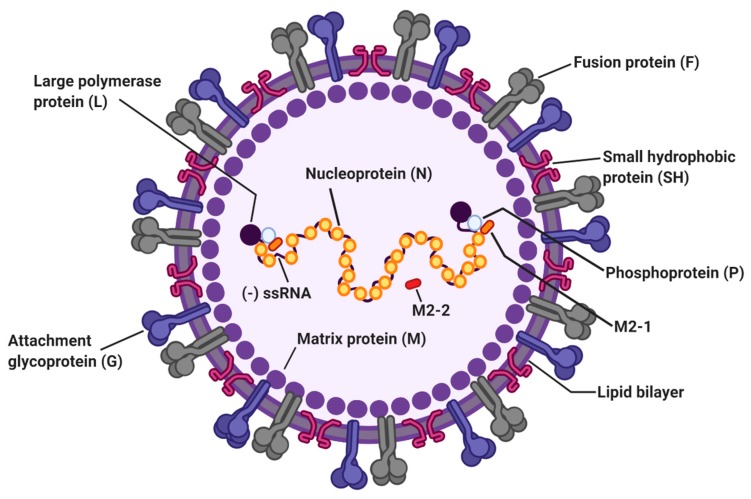
The structure of respiratory syncytial virus (RSV). The RSV genome is 15.2 kb of nonsegmented negative-sense RNA encoding 11 viral proteins. Viral envelope of RSV contains three transmembrane glycoproteins: attachment glycoprotein (G), fusion protein (F), and small hydrophobic protein (SH). Matrix proteins (M) are present on the inner side of the viral envelope. Viral RNA is tightly encapsidated by nucleoproteins (N) and the large proteins (L), phosphoproteins (P), and M2-1 proteins that mediate viral RNA transcription. M2-2 protein regulates viral RNA synthesis.

RSV is classified into subgroups A and B based on reactivity against monoclonal antibodies, with most differences occurring in the G protein [15]. A study demonstrated that subtype A is more virulent than subtype B [16]. The 15.2 kb RSV genome is a non-segmented negative-sense RNA encoding 11 viral proteins, namely nonstructural proteins NS1 and NS2, nucleoprotein (N), phosphoprotein (P), matrix protein (M), small hydrophobic protein (SH), attachment glycoprotein (G), fusion protein (F), M2-1, M2-2, and large protein (L) [17]. The RSV envelope contains three surface transmembrane glycoproteins, specifically G, F, and SH (Figure 1). Airway epithelial cells have been considered a primary target of RSV, with binding and entry of RSV into host cells mediated by the G and F proteins [18]. The G protein, which is expressed as soluble (Gs) and membrane-bound (Gm) forms, is responsible for viral attachment to host cells and immune modulation by RSV [19]. RSV entry is mediated by the F protein, which undergoes a conformational change and fuses the viral envelope with the host cell membrane [20]. Several candidate molecules have been proposed as an RSV receptor, including CX3 chemokine receptor 1 (CX3CR1) [21,22,23], DC-SIGN [24], heparan sulfate proteoglycans (HGPGs) [25], and annexin II [26]. The G protein contains a CX3C motif that can bind the CX3CR1 receptor on host cells; mutation of this motif or inhibition of the G-CX3CR1 interaction with a blocking anti-CX3CR1 antibody is reported to reduce RSV infection [23,27]. Recently, nucleolin was identified as a functional fusion receptor for RSV [28,29,30]. Silencing lung nucleolin using specific siRNA resulted in diminished RSV titers in infected mice, suggesting nucleolin as a functional cellular receptor for RSV [28]. The SH protein forms a pentameric ion channel that enhances membrane permeability in the host [31,32]. Studies demonstrated that deletion of SH in RSV leads to viral attenuation [33]. Although all three RSV surface proteins (F, G, and SH) are major targets of humoral immune responses, vaccine development for RSV has been focused primarily on the F protein, which is generally conserved across all known RSV strains [34]. Published reports have shown that F protein-specific antibodies induce the most neutralizing activity, suggesting a critical role for this protein [35,36]. M proteins, which are present on the interior side of the viral envelope, consist of a structural component and play an essential role in viral assembly and filament formation [37,38]. Viral RNA is tightly encapsidated by N proteins and the L, P, and M2-1 proteins that carry out viral RNA transcription [39]. The RSV M2-2 protein is involved in maintaining the balance between viral genome replication and transcription by negatively regulating viral transcription [40]. Although the non-structural proteins NS1 and NS2 do not directly participate in RNA replication, NS proteins facilitate RSV replication by disrupting type I IFN signaling in the host [41]. 

The Th1 and cytotoxic CD8^+^ T cell responses are both crucial for viral clearance and pathogenesis following RSV infection [42]. Moreover, RSV-specific neutralizing antibody responses confer protection against RSV infection [43]. It was reported that RSV-specific serum-neutralizing antibody levels were positively related to the resistance against RSV infection in adults [44] and the elderly [45], and the severity of RSV reinfection was inversely related to the titers of serum-neutralizing antibodies in children [46,47]. Further, passive transfer with Palivizumab, a humanized murine monoclonal neutralizing antibody to RSV F protein, achieved protection against infection with RSV in young children [48] indicating a protective role of antibodies during RSV infection. Interestingly, RSV-specific nasal IgA seems to be more effective than serum IgA to prevent RSV infection, but IgA+ memory B cells were undetectable at convalescence [49]. As nasal IgA is responsible for protection against RSV, inducing durable nasal IgA responses is considered an effective approach for RSV vaccine development. In addition, recent studies showed that passive administration of antibodies to RSV G protein also efficiently prevents RSV infection in mice, while treatment with the neutralizing antibody Palivizumab, which targets the F protein of RSV, is the only FDA-approved method for prevention of RSV infection [50,51]. Animal models [52,53,54,55] and human studies [56,57] of RSV infection demonstrated that Th2 cytokines (e.g., interleukin (IL)-4, IL-5, and IL-13) contribute to airway pathogenesis following RSV infection, suggesting that inappropriate activation of Th2 responses is harmful for RSV-infected hosts. Although there were several attempts to develop a safe and effective RSV vaccine, the potential candidates repeatedly failed to confer effective protection. Furthermore, some vaccine candidates caused enhanced respiratory disease, rather than protection, upon exposure to RSV. Studies conducted in 1966–1967 demonstrated that administration of formalin-inactivated RSV vaccines (FI-RSV) to infants and children resulted in severe respiratory disease upon subsequent natural RSV infection [58,59,60]. Hospitalization was required for 80% of the participants, and two vaccinated infants died upon infection, implying that primary immunization with FI-RSV induced aberrant pathologic responses. Indeed, subsequent studies on animal models revealed that FI-RSV boosted Th2-mediated immune responses [61,62,63].

Although an imbalance between Th1/Th2 immune responses accounts for the immunopathology during RSV infection, regulatory T cells (Tregs) are also essential for regulating a robust inflammatory response. Treg depletion leads to enhanced RSV disease accompanied by severe weight loss and delayed recovery [64,65,66]. Selective chemoattraction of Tregs to the airway by chemokine CCL17/22 administration ameliorated RSV vaccine-induced lung disease [67]. Consistent with these findings, injection of an IL-2/anti-IL-2 immune complex resulted in Treg accumulation and reduced lung inflammation following RSV infection [66], indicating that Tregs are responsible for controlling disease severity during RSV infection.

## 3. Innate Sensors Involved in RSV Recognition

**Figure 2:**
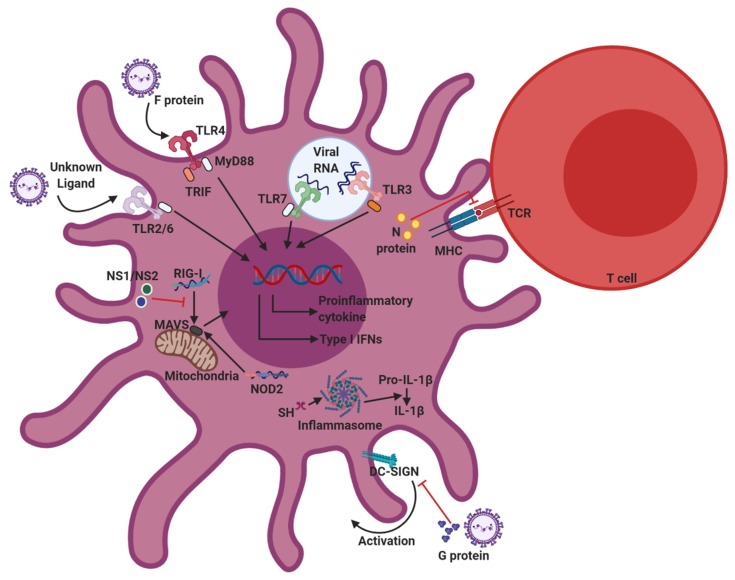
Innate sensors involved in RSV recognition, and immunomodulation strategies of RSV. Upon RSV infection, Toll-like receptors (TLR)2/6, TLR3, TLR4, TLR7, retinoic acid-inducible gene-I (RIG-I), and nucleotide-binding oligomerization domain (NOD2) are responsible for recognizing RSV pathogen-associated molecular patterns (PAMPs) in dendritic cells (DCs). The recognition of PAMPs by pattern recognition receptors (PRRs) activates downstream signaling pathways, which trigger DC activation and cytokine production. To avoid host immune responses, RSV has evolved various immunomodulatory strategies that inhibit DC functions. RSV proteins, specifically proteins G, NS1/NS2, and N, contribute to immunomodulation of RSV.

Host immune cells possess various pattern recognition receptors (PRRs), such as Toll-like receptors (TLRs), retinoic acid-inducible gene-I (RIG-I)-like receptors (RLRs), and nucleotide-binding oligomerization domain (NOD)-like receptors (NLRs), that initiate innate immune responses. PRRs recognize pathogen-associated molecular patterns (PAMPs) on pathogens to activate the production of proinflammatory cytokines and type I IFNs. Upon RSV infection, TLR2/6, TLR3, TLR4, TLR7, RIG-I, and NOD2 become important for recognizing RSV PAMPs (Figure 2). These PRRs are also expressed on DCs and associated with initiation of the innate immune response against viral infection [68]. 

### 3.1. TLR Signaling

TLRs are essential for activation of innate immune responses by recognizing PAMPs derived from various pathogens. TLR signaling is mediated though the specific adaptor molecules that activate NF-κB and IRFs and leads to subsequent initiation of innate immune responses including production of cytokines and type I IFNs. MyD88 is a downstream adaptor protein involved in signaling from TLRs except TLR3. MyD88 deficiency abrogated IFN-β secretion from bone marrow (BM)-DCs following RSV infection, even though TLR7 was dispensable for RSV-induced IFN-β production [69]. BM-DCs from MyD88-deficient mice also displayed impairment of IL-12 production during RSV infection [70].

The first PRR that was determined to be activated by RSV infection was TLR4. While TLR4 is well-known for recognizing lipopolysaccharide (LPS) on Gram-negative bacteria, the RSV F protein also induces TLR4 activation [71,72,73]. Furthermore, RSV infection enhances TLR4 expression on epithelial cells and monocytes [74,75]. RSV-induced TLR4 activation led to NF-κB mediated proinflammatory cytokine production [76], and macrophages isolated from TLR4-deficient mice displayed abolished IL-6 production following RSV F protein stimulation [71]. TLR4 polymorphisms were implicated in determining susceptibility to RSV infection [77,78] and RSV viral clearance was delayed in TLR4-deficient mice [71,72,79]. 

TLR2/6 is an extracellular receptor that recognizes microbial cell wall components. The specific mechanisms involved in RSV sensing via TLR2/6 are unclear. Nevertheless, studies have shown that TLR2/6 is activated during RSV infection and induces NF-κB-driven pro-inflammatory cytokine production though the MyD88 pathway [79]. In addition, RSV infection leads to upregulation of TLR2 expression [80]. Moreover, either TLR2- or TLR6-deficient macrophages displayed reduced pro-inflammatory cytokine production (including IL-6 and TNF-α) following RSV infection, and TLR2 or TLR6 knockout mice showed increased viral load and a reduced number of activated DCs in bronchoalveolar lavage (BAL) samples following RSV infection. Collectively, these findings demonstrate that TLR2/6 signaling is involved in the host defense against RSV [79]. 

TLR7 is an endosomal single-stranded RNA (ssRNA) receptor that initiates type I IFN and proinflammatory cytokine production via the MyD88-mediated pathway. RSV infection upregulates TLR7 expression in the lungs. Although RSV-induced IFN-β production from bone marrow-derived DCs (BM-DCs) and macrophages (BM-DMs) is independent of the TLR7 pathway [69], plasmacytoid dendritic cell (pDC)-derived IFN-β production is dependent on TLR7-MyD88 in mice [81]. In addition, TLR7 plays an important role in T cell polarization during RSV infection. Th1-promoting IL-12 levels were shown to be diminished in TLR7-deficient BM-DCs, while Th17-promoting IL-23 expression increased following RSV infection. TLR7-deficient mice displayed exacerbated airway pathological features accompanied by mucus hypersecretion and overexpression of IL-4, IL-13, and IL-17 in the lungs [82].

TLR3, an intracellular receptor localized in endosomes, is commonly regarded as a double-stranded RNA (dsRNA) sensor. RSV increases TLR3 expression and immune responses to dsRNA in host cells [83]. RSV-infected TLR3-deficient mice showed increased mucus production and pulmonary IL-13 and IL-5 expression in the airway even though they did not display defects in viral clearance, demonstrating that TLR3 deficiency during RSV infection skews host immunity toward a Th2-mediated response [84]. 

These studies suggest that the TLR signaling pathway promotes the production of proinflammatory cytokines and type I IFNs in DCs following RSV infection. Further investigation is still required to better understand the underlying mechanisms in greater detail.

### 3.2. RIG-I and MAVS

RIG-I is an intracellular PRR that senses 5′-triphosphorylated viral RNA in the cytosol. RIG-I detects 5′-triphosphate through its helicase domain and interacts with the mitochondrial anti-viral signaling protein (MAVS, also known as IFN-β promoter stimulator 1), which induces proinflammatory cytokines and type I IFN production via IRF3/7 and NF-κB activation. RSV infection induces RIG-I expression in epithelial cells, and RIG-I recognizes RSV transcripts as a ligand [85]. Mouse fibroblasts lacking RIG-I showed attenuated expression of IRF-3-dependent genes including ISG15, ISG54, and ISG56 [86]. Moreover, siRNA-mediated RIG-I silencing in epithelial cells decreased IRF3- and NF-κB-mediated IFN-β, IP-10, CCL5, and ISG15 expression following RSV infection [85]. Indeed, RSV-induced IFN-β production in BM-DCs and BM-DMs was abrogated following genetic ablation of MAVS [69], and MAVS deficiency resulted in severe inflammation accompanied by reduced BAL fluid IFN-β levels in RSV-infected animals [87]. Consistent with this result, cDCs isolated from Mavs^−/−^ mice were unable to produce type I IFNs during RSV infection. RSV-induced proinflammatory cytokine and type I IFN levels in BAL fluid were also abrogated in these animals, suggesting a dependency on MAVS [88]. 

### 3.3. NOD2 and Inflammasomes

NOD2 is a cytoplasmic molecule that detects ssRNA. Recognition of the RSV ssRNA genome by NOD2 initiates type I IFN production via IRF3 activation. RSV-infected epithelial cells express NOD2 within 2 h after infection and siRNA-mediated knockdown of NOD2 reduced activation of IRF3 and IFN-β production in RSV-infected epithelial cells [89]. Consistent with this finding, NOD2-deficiency abrogated IFN-β production in the respiratory tract of RSV-infected mice. Data demonstrating uncontrolled RSV titers and severe lung pathology in NOD-deficient, RSV-infected mice demonstrate that NOD2 is a critical component in triggering anti-RSV responses in hosts.

The inflammasome is a multimeric intracellular protein complex responsible for the activation of inflammatory responses. Certain NOD-like receptors trigger assembly of the inflammasome complex. Moreover, caspase-1 activation leads to the production of proinflammatory cytokines IL-1β and IL-18, as well as programmed cell death. While specific mechanisms have yet to be fully elucidated, one study reported that RSV infection causes IL-1β release via NLRP3/ASC inflammasome activation and that caspase-1 activation is inhibited by NLRP3 or ASC deficiency [90]. Further, infection with an SH-deficient mutant RSV failed to produce IL-1β from lung epithelial cells, indicating that SH is responsible for inflammasome activation and IL-1β production during RSV infection [91].

Several studies suggest that PRRs play an important role in initiating immune responses to RSV infection. Activated PRRs that detect RSV-derived viral components are implicated in the production of type I IFN and proinflammatory cytokines, as well as T cell response skewing. Consistently, MyD88/Trif/Mavs^−/−^ (MTM^−/−^) mice that cannot induce PRR-mediated signaling pathways failed to produce innate cytokines such as IFN-α, IL-6, and IL-1β [92]. Despite the fact that MTM^−/−^ mice were still able to elicit RSV-specific CD8^+^ T cell responses, they showed enhanced susceptibility to RSV infection accompanied by impaired viral control and severe weight loss. Furthermore, anti-RSV antibody production was significantly reduced in MyD88^−/−^ mice, Mavs^−/−^ mice, and MyD88^−/−^Mavs^−/−^ double knockout mice [88]. These findings indicate that PRR-mediated signaling is crucial for regulating disease severity in RSV-infected hosts. The specific roles of each PRR in DCs are not yet fully clarified. Therefore, further DC-specific studies are required to understand the contribution of PRRs to anti-RSV responses in DCs.

## 4. Lung Resident DCs and RSV Infection

Over the years, researchers have demonstrated that adaptive responses are responsible for efficient viral clearance following RSV infection and RSV-induced pathology. As DCs are professional antigen-presenting cells that mediate T cell activation by antigen presentation, various approaches were attempted to elucidate the role of DCs in adaptive immunity against RSV infection. 

DCs are specialized cells that play an essential role in linking innate and adaptive immune responses. Following RSV infection, DCs acquire viral antigens directly through infection or indirectly by phagocytosis of virus-infected cells. DCs express PRRs and the recognition of PAMPs by PRRs activates downstream signaling pathways, which trigger DC maturation and cytokine production [93]. Consequently, RSV exposure promotes maturation of DC subsets, including those infected directly and uninfected ones in which costimulatory molecules such as CD80 and CD86 are upregulated [94,95,96,97,98]. RSV infection also promotes MHC class II expression [99] and induces the production of proinflammatory cytokines and type I IFNs from DCs [95,100]. DCs that capture viral antigens migrate to the draining lymph nodes to initiate and organize viral-specific T cell responses by presenting antigen peptides bound to class I or class II MHC molecules [101].

**Figure 3:**
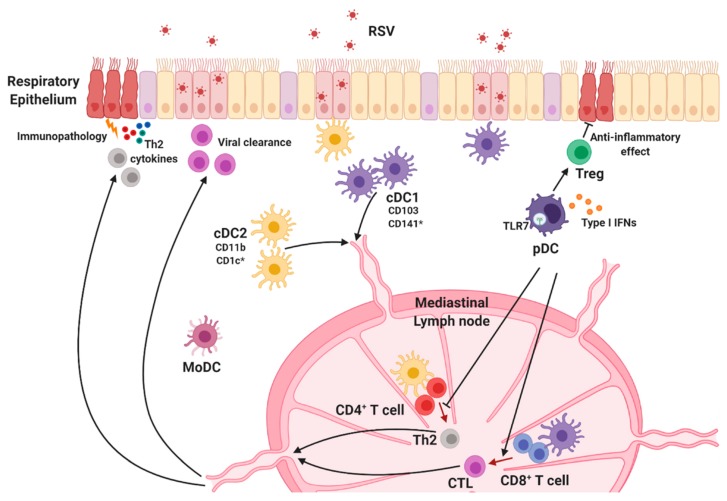
Lung dendritic cell subsets. Lung DCs are classified into conventional DC1s (cDC1s), cDC2s, and plasmacytoid DCs (pDCs). Each DC subset is widely distributed throughout the lungs and migrates to the lung-draining lymph node when they recognize RSV to initiate protective immune responses. cDC1s preferentially activate CD8^+^ T cells that mediate viral clearance, and cDC2s are responsible for Th2-mediated immune responses and RSV-mediated pulmonary diseases. pDCs are the main source of type I interferons (IFNs) and play an essential role in RSV-specific cytotoxic T lymphocyte (CTL) priming and regulation of disease severity. * Human-specific marker.

Lung DCs are classified into cDCs and pDCs in the steady state, and monocyte-derived DCs (MoDCs) are generated after exposure to inflammatory stimulation (Figure 3) [102,103,104,105]. cDC subsets are defined in terms of cell surface marker expression and their ability to induce adaptive immune responses. Specifically, cDCs expressing CD11c^hi^-MHC class II^+^ are subdivided into CD103^+^ cDC1 and CD11b^+^ cDC2 groups in mice and CD141^+^ (BDCA-3) cDC1 and CD1c^+^ (BDCA-1) cDC2 groups in humans [106]. 

CD103^+^ cDC1s are functionally similar to CD8^+^ cDC1s in lymphoid organs [107]. Several studies revealed that cDC1 subsets preferentially activate CD8^+^ T cells via cross-presentation of antigens with MHC class I. cDC1s are also required for Th1 differentiation because they serve as a major producer of IL-12 [14,108,109,110,111,112]. Batf3^−/−^ mice, which lack CD103^+^ cDC1s [113], were used to elucidate the indispensable role of cDC1s in the development of CD8^+^ T cell responses to multiple pathogens. RSV-specific CD8^+^ T cells in the lungs were considerably reduced in adult Batf3^−/−^ mice, suggesting that anti-RSV CD8^+^ T cell responses in adult mice are dependent on CD103^+^ cDC1s [114]. In addition, studies on RSV-infected neonatal mice revealed that lung cDC1 populations in neonates are functionally defective compared with adults [114,115]. Neonatal DC responses against RSV are conducted primarily by CD103^+^ cDC1s that display deficiencies in the uptake and processing of a soluble antigen, as well as the expression of both CD80 and CD86 costimulatory molecules, following RSV infection [115]. Consistently, cDC1s in RSV-infected neonates showed early impairment in the ability to stimulate RSV K^d^M2_82–90_ epitope-targeting CD8^+^ T cells, indicating that their limited ability resulted in a different CD8^+^ T cell response hierarchy between neonates and adults [114,115,116]. Interestingly, Ruckwardt et al. reported that CD103^+^ cDC1s in RSV-infected neonates were composed of two phenotypically and functionally distinct populations, CD103^lo^ DCs and CD103^hi^ DCs, characterized by CD103 expression [114]. The authors demonstrated that CD103^lo^ DCs were immature, underdeveloped, and unable to stimulate the K^d^M2_82–90_-specific CD8^+^ T cell response, while CD103^hi^ DCs induced proliferation of both K^d^M2_82–90_-specific cells and D^b^M_187–195_-specific CD8^+^ T cells. Nevertheless, CD103^hi^ DCs seem to be different from adult CD103^+^ DCs because adult CD103^+^ cDC1s are more functional than either neonatal population. In addition, transforming growth factor beta (TGF-β) is suggested as regulator of DC-T cell responses against RSV [117]. Adult DCs isolated from peripheral blood produced lower TGF-β than cord blood DCs following RSV infection, and the addition of TGF-β changed cytokine secretion profiles reducing Th1 cytokines IFN-γ, TNF-α, and IL-2 productions during adult DC and T cell cocultures. These findings suggest cellular mechanisms to understand functional differences between adult and neonatal DCs.

Unlike cDC1s, cDC2 subsets are considered to be responsible for the activation of CD4^+^ T cells by MHC-II-dependent antigen presentation [118]. Consequently, these subsets contribute to helper T cell polarization, including differentiation into Th2, Th17, and T follicular helper cells [10,102,119,120,121]. These findings suggest the possibility that cDC2 populations support antibody-mediated protection. While specific roles of cDC2s in RSV infection have not been fully clarified yet, studies suggest that CD11b^+^ cDC2s mediate RSV-specific T cell responses. In mice, CCR6-expressing CD11b^+^ cDC2s are responsible for the induction of Th2 responses and Th2-mediated lung pathology during RSV infection [122]. Levels of CCL20, the ligand of CCR6, in the lungs are reportedly elevated following RSV infection, and accumulation of CCR6-expressing CD11b^+^ cDC2s is reduced in anti-CCL20-treated mice as well as in CCR6-deficient mice, demonstrating that CCL20/CCR6 plays a key role in recruiting CD11b^+^ cDC2s during RSV infection. Furthermore, absence of the CCL20/CCR6 axis led to ameliorated lung pathology accompanied by reduced Th2 cytokine and mucus production. Consistent with these results, an RSV infection model in neonates suggests that lung CD11b^+^ cDC2s promote Th2-biased immune responses in response to RSV infection [123]. Neonatal CD11b^+^ cDC2s displayed significantly higher levels of IL-4Rα than their adult counterparts, and IL-4Rα expression on CD11b^+^ cDC2s diminished with age. As expected, IL-4Rα-deficient CD11b^+^ cDC2s isolated from RSV-infected neonates showed an impaired capacity to induce Th2 responses, and CD11c^+^ cell-specific deletion of IL-4Rα protected mice from lung damage during RSV infection. These data are consistent with previous results showing that local inhibition of IL-4Rα expression using antisense oligonucleotides in neonates prevented pulmonary dysfunction following RSV infection [124]. Moreover, overexpression of IL-4Rα on CD11b^+^ adult cDC2s induced robust Th2 cytokine production in RSV-infected adult mice [123].

pDCs are known as the main source of type I IFNs. RSV infection results in the upregulation of MHC class II and CD80/CD86 costimulatory molecule expression, as well as the promotion of type I IFN production in pDCs [95,125,126,127]. Early studies on RSV demonstrated that pDCs play a crucial role in regulating viral replication, clearance, and immunopathology following RSV infection [125,128]. pDC depletion using the 120G8 antibody increased pulmonary RSV titers in mice [125,128], and adoptive transfer of bone marrow-derived pDCs reduced viral mRNA expression in RSV-infected lung tissue [125], indicating that pDCs regulate viral titers in RSV-infected mice. Interestingly, Flt3 ligand-induced expansion of DC subsets protects the host from severe RSV-induced immunopathology, which is dependent upon pDCs [127]. Pre-treatment of Flt3 ligand diminished harmful RSV-induced Th2 responses and increased CD8^+^ T cell responses to RSV; however, selective depletion of pDCs exacerbated lung inflammation and hampered RSV-specific CD8^+^ T cell responses in Flt3 ligand-treated mice [127]. Furthermore, diphtheria toxin-induced pDC depletion in BDCA2-DTR mice led to attenuated cytotoxic T lymphocyte (CTL) responses against RSV infection [81]. Both studies consistently reported that pDCs are required for RSV-specific CTL priming. In addition, mice that had undergone antibody-mediated pDC depletion exhibited severe pulmonary inflammation, airway hyper-reactivity, and mucus production following RSV infection [125,128]. Remarkably, mRNA expression levels of Th2 cytokines, which contribute to airway pathogenesis, were significantly increased in the lungs of pDC-depleted mice during RSV infection. Th2 cytokine production by lymph node T cells was also enhanced in these pDC-depleted mice [128]. Studies of neonatal mouse models of RSV reinfection demonstrated that RSV-infected neonates produced significantly lower levels of type I IFNs along with reduced pDC recruitment in lungs compared with adults. IFN-α treatment or adoptive transfer of adult pDCs prior to neonatal RSV infection attenuated Th2-biased immune responses and lung pathology upon RSV reinfection in adult animals [129]. These findings suggest that pDCs are associated with regulating harmful Th2-type responses and Th2-mediated immunopathology following RSV infection. Recently, a study using a murine model of pneumonia virus of mice (PVM), a mouse-specific pneumovirus related to RSV, revealed that surface expression of Semaphorin 4a (Sema 4a), the ligand for Nrp-1, by pDCs contributes to the expansion of Nrp-1^+^ Tregs that prevent the development of PVM-induced bronchiolitis in early life and subsequent asthma following reinfection [130]. pDC depletion or Sema4a blockade in neonates abrogated Treg expansion during PVM infection, as well severe bronchiolitis and asthma in later life, suggesting that pDCs limit PVM-induced immunopathology by expanding Tregs [130]. Taken together, these studies demonstrate that pDCs play an essential role in inducing adaptive T cell responses that protect the host from severe RSV disease.

MoDCs constitute a heterogeneous group of cells derived from monocytes under inflammatory conditions [14]. Since both cell subsets express CD11b, it is difficult to distinguish MoDCs from CD11b^+^ cDC2s. Although studies examining the role of MoDCs during RSV infection are limited, data from in vitro studies suggest that MoDCs are very susceptible to RSV [95,131]. Productive infection of MoDCs by RSV upregulated the expression of maturation makers such as CD83, CD86, and MHC molecules on these cells; however, the ability of MoDCs to induce naïve CD4^+^ T cell activation and proliferation was inefficient [95,96]. While the specific mechanisms underlying their role remain unknown, these findings suggest that MoDCs are implicated in the immunomodulation of RSV. 

## 5. Immune Evasion Strategies of RSV That Modulate DC Function

RSV utilizes several mechanisms to evade the host immune system by disrupting both the innate and adaptive immune responses. RSV proteins, specifically proteins G, NS1/NS2, and N, contribute to immunomodulation of RSV. These proteins interfere with host immune components to promote viral propagation (Figure 2). 

As described above, RSV G proteins are expressed as both membrane-anchored (mG protein) and soluble forms (sG protein) [132]. G protein binds to the CX3C chemokine receptor CX3CR1 on host cells through the CX3C motif, and therefore competes with CX3CL1 to induce leukocyte chemotaxis as well as facilitate RSV infection [21]. Regarding DCs, DC-SIGN and L-SIGN are binding targets for RSV G protein [24]. Interactions between G protein and DC- or L-SIGN inhibit DC activation and the production of cytokines including IFN-α, MIP-1α, and MIP-1β from DCs. Interestingly, these interactions are not associated with productive RSV infection [24]. Finally, G protein downregulates IFN-α and TNF-α production in pDCs [133], plus IFN-β production in mDCs [19], indicating that the RSV G protein alone yields insufficient immune responses against RSV infection in DCs. 

Studies have established that the NS1 and NS2 non-structural proteins suppress type I IFN production and signaling in RSV-infected cells. NS1 and NS2 proteins inhibit anti-RSV responses in the host both independently and in conjunction with each other [41]. Early studies on recombinant RSV lacking NS1 and NS2 demonstrated that NS1/NS2 deficiency induces attenuated viral replication [33,41,134,135]. Specifically, NS1 and NS2 interfere with the RIG-I/MAVS-dependent type I IFN production pathway. NS1 binds to MAVS [136], while NS2 targets the N-terminal CARD of RIG-I [137] to disrupt RIG-I/MAVS interactions and inhibits downstream activation of IRF-3, which initiates transcription of type I IFN genes [134,138]. NS1 also directly interacts with IRF-3, thereby preventing IRF-3 binding to the *IFN-β* promoter region [139]. Indeed, NS1 and NS2 decrease type I IFN responsiveness in host cells by inhibiting STAT2, a transcription factor downstream of the type I IFN receptor [140,141,142,143]. Both NS1 and NS2 elicit ubiquitination and proteasomal degradation of STAT2. In RSV-infected DCs, NS1 and NS2 mediate the negative modulation of DC maturation [144]. In addition to regulating type I IFN production, NS1/NS2 suppress the surface expression of maturation markers, including CD80, CD86, and CD38, on DCs [144], and control the ability of DCs to activate T cells. NS1 promotes DCs to induce pathogenic Th2-biased CD4^+^ T cell responses and inhibits the activation of CD8^+^ T cells that express the tissue homing integrin CD103 [145]. Overall, NS1/NS2 suppress the ability of DCs to activate protective T cell responses. 

The RSV N protein also possesses immunomodulatory properties. RSV prevents T cell activation by disrupting DC-T cell synapse assembly, and N protein plays a role in this inhibitory process [146,147]. Early in vitro studies on RSV-infected BM-DCs showed that the interaction between RSV-infected DCs and T cells results in unresponsiveness to TCR stimuli by T cells due to impaired formation of the immunological synapse [146]. While the specific mechanisms are unclear, surface-expressed N protein on RSV-infected DCs accumulates at the synaptic center with the TCR complex, inhibiting MHC–TCR interactions [147].

Interestingly, RSV seems to manipulate gene expression in host cells through microRNA [148,149]. In monocyte-derived DCs, let-7b expression was upregulated following RSV infection while let-7i and miR-30b were upregulated in NHBE human bronchial epithelial cells [148]. RSV-infected A549 human alveolar epithelial cells displayed changed microRNA expression profiles including let-7f [149]. While RSV G protein [149] and NS1/2 proteins [148] appear to be associated with the regulation of miRNA expression, further studies are needed to elucidate the role of miRNA in host immune responses. 

## 6. Conclusions

RSV infection is a leading cause of severe respiratory disease and hospitalization in infants, as well as children. Most people experience their initial RSV infection by two years of age [47] and RSV reinfection occurs throughout life. While RSV reinfection causes mild symptoms in healthy adults, elderly and immunocompromised individuals have high morbidity and mortality risk. Due to the health burden of RSV, several approaches were attempted to develop an effective vaccine to prevent RSV infection. In the 1960s, the first RSV vaccine candidate FI-RSV failed to establish suitable anti-RSV immune responses. Instead, a fatal respiratory illness following natural RSV infection was elicited. Since then, the goals for RSV vaccine development involve prevention of both viral infection and serious adverse side effects. However, previous RSV vaccine strategies were unsuccessful, and a licensed vaccine remains available currently. Palivizumab, a humanized monoclonal neutralizing antibody targeting the F protein of RSV, is the first and only FDA-approved agent for the prevention of RSV infection. While prophylactic treatment with Palivizumab prevents viral infection effectively [48], this therapeutic is expensive and thus recommended only for infants who are at high risk. Therefore, additional investigation is still required to develop a safe and effective vaccine, as well as therapeutics for RSV infection.

Since DCs play an essential role in establishing both protective and pathogenic immune responses following RSV infection, understanding the specific mechanisms of how these cells recognize RSV and initiate adaptive immune responses, as well as how RSV inhibits DC functions to avoid host defensive tactics, will provide insight into strategies for anti-RSV therapy and vaccine development. Interestingly, TLR-agonist treatment at the time of RSV infection increased RSV-specific CD8^+^ T cells in neonates via upregulation of CD86 expression on cDCs, indicating that DCs can be potential targets of anti-RSV therapy [150]. Recently, a novel protein-based RSV vaccine was reported [151,152]. The researchers developed the DS-Cav1 vaccine, which targets the pre-fusion form of RSV F protein, and vaccination with DS-Cav1 elicited superior neutralizing antibody responses in healthy adult volunteers [152]. Increasing vaccine immunogenicity by fusing DS-Cav1 to nanoparticles was reported to induce more robust humoral responses than trimeric DS-Cav1 [153]. Thus, combining DC-targeting therapy with vaccination could potentially produce an additive or synergistic effect that ultimately treats RSV infection with minimal adverse effects.

## References

[1] Afonso C.L., Amarasinghe G.K., Banyai K., Bao Y., Basler C.F., Bavari S., Bejerman N., Blasdell K.R., Briand F.X., Briese T. (2016). Taxonomy of the order Mononegavirales: Update 2016. Arch. Virol..

[2] Blount R.E., Morris J.A., Savage R.E. (1956). Recovery of cytopathogenic agent from chimpanzees with coryza. Proc. Soc. Exp. Biol. Med..

[3] Chanock R., Finberg L. (1957). Recovery from infants with respiratory illness of a virus related to chimpanzee coryza agent (CCA). II. Epidemiologic aspects of infection in infants and young children. Am. J. Hyg..

[4] Chanock R., Roizman B., Myers R. (1957). Recovery from infants with respiratory illness of a virus related to chimpanzee coryza agent (CCA). I. Isolation, properties and characterization. Am. J. Hyg..

[5] Mitchell I., Defoy I., Grubb E. (2017). Burden of Respiratory Syncytial Virus Hospitalizations in Canada. Can. Respir. J..

[6] Amand C., Tong S., Kieffer A., Kyaw M.H. (2018). Healthcare resource use and economic burden attributable to respiratory syncytial virus in the United States: A claims database analysis. BMC Health Serv. Res..

[7] Hall C.B., Walsh E.E., Long C.E., Schnabel K.C. (1991). Immunity to and frequency of reinfection with respiratory syncytial virus. J. Infect. Dis..

[8] Ruckwardt T.J., Morabito K.M., Graham B.S. (2019). Immunological Lessons from Respiratory Syncytial Virus Vaccine Development. Immunity.

[9] Wang H., Griffiths M.N., Burton D.R., Ghazal P. (2000). Rapid antibody responses by low-dose, single-step, dendritic cell-targeted immunization. Proc. Natl. Acad. Sci. USA.

[10] Krishnaswamy J.K., Gowthaman U., Zhang B., Mattsson J., Szeponik L., Liu D., Wu R., White T., Calabro S., Xu L. (2017). Migratory CD11b(+) conventional dendritic cells induce T follicular helper cell-dependent antibody responses. Sci. Immunol..

[11] Shin C., Han J.A., Koh H., Choi B., Cho Y., Jeong H., Ra J.S., Sung P.S., Shin E.C., Ryu S. (2015). CD8alpha(-) Dendritic Cells Induce Antigen-Specific T Follicular Helper Cells Generating Efficient Humoral Immune Responses. Cell Rep..

[12] Tesfaye D.Y., Gudjonsson A., Bogen B., Fossum E. (2019). Targeting Conventional Dendritic Cells to Fine-Tune Antibody Responses. Front. Immunol..

[13] Granot T., Senda T., Carpenter D.J., Matsuoka N., Weiner J., Gordon C.L., Miron M., Kumar B.V., Griesemer A., Ho S.H. (2017). Dendritic Cells Display Subset and Tissue-Specific Maturation Dynamics over Human Life. Immunity.

[14] Eisenbarth S.C. (2019). Dendritic cell subsets in T cell programming: Location dictates function. Nat. Rev. Immunol..

[15] Mufson M.A., Orvell C., Rafnar B., Norrby E. (1985). Two distinct subtypes of human respiratory syncytial virus. J. Gen. Virol..

[16] Jafri H.S., Wu X., Makari D., Henrickson K.J. (2013). Distribution of respiratory syncytial virus subtypes A and B among infants presenting to the emergency department with lower respiratory tract infection or apnea. Pediatr. Infect. Dis. J..

[17] Atreya P.L., Peeples M.E., Collins P.L. (1998). The NS1 protein of human respiratory syncytial virus is a potent inhibitor of minigenome transcription and RNA replication. J. Virol..

[18] Zhang L., Peeples M.E., Boucher R.C., Collins P.L., Pickles R.J. (2002). Respiratory syncytial virus infection of human airway epithelial cells is polarized, specific to ciliated cells, and without obvious cytopathology. J. Virol..

[19] Shingai M., Azuma M., Ebihara T., Sasai M., Funami K., Ayata M., Ogura H., Tsutsumi H., Matsumoto M., Seya T. (2008). Soluble G protein of respiratory syncytial virus inhibits Toll-like receptor 3/4-mediated IFN-beta induction. Int. Immunol..

[20] Gilman M.S.A., Furmanova-Hollenstein P., Pascual G., van’t Wout A.B., Langedijk  J.P.M., McLellan J.S. (2019). Transient opening of trimeric prefusion RSV F proteins. Nat. Commun..

[21] Tripp R.A., Jones L.P., Haynes L.M., Zheng H., Murphy P.M., Anderson L.J. (2001). CX3C chemokine mimicry by respiratory syncytial virus G glycoprotein. Nat. Immunol..

[22] Harcourt J., Alvarez R., Jones L.P., Henderson C., Anderson L.J., Tripp R.A. (2006). Respiratory syncytial virus G protein and G protein CX3C motif adversely affect CX3CR1+ T cell responses. J. Immunol..

[23] Chirkova T., Lin S., Oomens A.G., Gaston K.A., Boyoglu-Barnum S., Meng J., Stobart C.C., Cotton C.U., Hartert T.V., Moore M.L. (2015). CX3CR1 is an important surface molecule for respiratory syncytial virus infection in human airway epithelial cells. J. Gen. Virol..

[24] Johnson T.R., McLellan J.S., Graham B.S. (2012). Respiratory syncytial virus glycoprotein G interacts with DC-SIGN and L-SIGN to activate ERK1 and ERK2. J. Virol..

[25] Krusat T., Streckert H.J. (1997). Heparin-dependent attachment of respiratory syncytial virus (RSV) to host cells. Arch. Virol..

[26] Malhotra R., Ward M., Bright H., Priest R., Foster M.R., Hurle M., Blair E., Bird M. (2003). Isolation and characterisation of potential respiratory syncytial virus receptor(s) on epithelial cells. Microbes Infect..

[27] Boyoglu-Barnum S., Todd S.O., Meng J., Barnum T.R., Chirkova T., Haynes L.M., Jadhao S.J., Tripp R.A., Oomens A.G., Moore M.L. (2017). Mutating the CX3C Motif in the G Protein Should Make a Live Respiratory Syncytial Virus Vaccine Safer and More Effective. J. Virol..

[28] Tayyari F., Marchant D., Moraes T.J., Duan W., Mastrangelo P., Hegele R.G. (2011). Identification of nucleolin as a cellular receptor for human respiratory syncytial virus. Nat. Med..

[29] Holguera J., Villar E., Munoz-Barroso I. (2014). Identification of cellular proteins that interact with Newcastle Disease Virus and human Respiratory Syncytial Virus by a two-dimensional virus overlay protein binding assay (VOPBA). Virus Res..

[30] Mastrangelo P., Hegele R.G. (2013). RSV fusion: Time for a new model. Viruses.

[31] Gan S.W., Ng L., Lin X., Gong X., Torres J. (2008). Structure and ion channel activity of the human respiratory syncytial virus (hRSV) small hydrophobic protein transmembrane domain. Protein Sci..

[32] Gan S.W., Tan E., Lin X., Yu D., Wang J., Tan G.M., Vararattanavech A., Yeo C.Y., Soon C.H., Soong T.W. (2012). The small hydrophobic protein of the human respiratory syncytial virus forms pentameric ion channels. J. Biol. Chem..

[33] Whitehead S.S., Bukreyev A., Teng M.N., Firestone C.Y., St Claire M., Elkins W.R., Collins P.L., Murphy B.R. (1999). Recombinant respiratory syncytial virus bearing a deletion of either the NS2 or SH gene is attenuated in chimpanzees. J. Virol..

[34] Hause A.M., Henke D.M., Avadhanula V., Shaw C.A., Tapia L.I., Piedra P.A. (2017). Sequence variability of the respiratory syncytial virus (RSV) fusion gene among contemporary and historical genotypes of RSV/A and RSV/B. PLoS ONE.

[35] Magro M., Mas V., Chappell K., Vazquez M., Cano O., Luque D., Terron M.C., Melero J.A., Palomo C. (2012). Neutralizing antibodies against the preactive form of respiratory syncytial virus fusion protein offer unique possibilities for clinical intervention. Proc. Natl. Acad. Sci. USA.

[36] Ngwuta J.O., Chen M., Modjarrad K., Joyce M.G., Kanekiyo M., Kumar A., Yassine H.M., Moin S.M., Killikelly A.M., Chuang G.Y. (2015). Prefusion F-specific antibodies determine the magnitude of RSV neutralizing activity in human sera. Sci. Transl. Med..

[37] Mitra R., Baviskar P., Duncan-Decocq R.R., Patel D., Oomens A.G. (2012). The human respiratory syncytial virus matrix protein is required for maturation of viral filaments. J. Virol..

[38] Ghildyal R., Mills J., Murray M., Vardaxis N., Meanger J. (2002). Respiratory syncytial virus matrix protein associates with nucleocapsids in infected cells. J. Gen. Virol..

[39] Tran T.L., Castagne N., Dubosclard V., Noinville S., Koch E., Moudjou M., Henry C., Bernard J., Yeo R.P., Eleouet J.F. (2009). The respiratory syncytial virus M2-1 protein forms tetramers and interacts with RNA and P in a competitive manner. J. Virol..

[40] Bermingham A., Collins P.L. (1999). The M2-2 protein of human respiratory syncytial virus is a regulatory factor involved in the balance between RNA replication and transcription. Proc. Natl. Acad. Sci. USA.

[41] Spann K.M., Tran K.C., Chi B., Rabin R.L., Collins P.L. (2004). Suppression of the induction of alpha, beta, and lambda interferons by the NS1 and NS2 proteins of human respiratory syncytial virus in human epithelial cells and macrophages [corrected]. J. Virol..

[42] Graham B.S., Bunton L.A., Wright P.F., Karzon D.T. (1991). Role of T lymphocyte subsets in the pathogenesis of primary infection and rechallenge with respiratory syncytial virus in mice. J. Clin. Investig..

[43] Graham B.S., Bunton L.A., Rowland J., Wright P.F., Karzon D.T. (1991). Respiratory syncytial virus infection in anti-mu-treated mice. J. Virol..

[44] Luchsinger V., Piedra P.A., Ruiz M., Zunino E., Martinez M.A., Machado C., Fasce R., Ulloa M.T., Fink M.C., Lara P. (2012). Role of neutralizing antibodies in adults with community-acquired pneumonia by respiratory syncytial virus. Clin. Infect. Dis..

[45] Walsh E.E., Peterson D.R., Falsey A.R. (2004). Risk factors for severe respiratory syncytial virus infection in elderly persons. J. Infect. Dis..

[46] Kawasaki Y., Hosoya M., Katayose M., Suzuki H. (2004). Role of serum neutralizing antibody in reinfection of respiratory syncytial virus. Pediatr. Int..

[47] Glezen W.P., Taber L.H., Frank A.L., Kasel J.A. (1986). Risk of primary infection and reinfection with respiratory syncytial virus. Am. J. Dis. Child..

[48] Feltes T.F., Cabalka A.K., Meissner H.C., Piazza F.M., Carlin D.A., Top F.H., Connor E.M., Sondheimer H.M., Cardiac Synagis Study G. (2003). Palivizumab prophylaxis reduces hospitalization due to respiratory syncytial virus in young children with hemodynamically significant congenital heart disease. J. Pediatr..

[49] Habibi M.S., Jozwik A., Makris S., Dunning J., Paras A., DeVincenzo J.P., de Haan C.A., Wrammert J., Openshaw P.J., Chiu C. (2015). Impaired Antibody-mediated Protection and Defective IgA B-Cell Memory in Experimental Infection of Adults with Respiratory Syncytial Virus. Am. J. Respir. Crit. Care Med..

[50] Boyoglu-Barnum S., Todd S.O., Chirkova T., Barnum T.R., Gaston K.A., Haynes L.M., Tripp R.A., Moore M.L., Anderson L.J. (2015). An anti-G protein monoclonal antibody treats RSV disease more effectively than an anti-F monoclonal antibody in BALB/c mice. Virology.

[51] Han J., Takeda K., Wang M., Zeng W., Jia Y., Shiraishi Y., Okamoto M., Dakhama A., Gelfand E.W. (2014). Effects of anti-g and anti-f antibodies on airway function after respiratory syncytial virus infection. Am. J. Respir. Cell Mol. Biol..

[52] Lukacs N.W., Moore M.L., Rudd B.D., Berlin A.A., Collins R.D., Olson S.J., Ho S.B., Peebles R.S. (2006). Differential immune responses and pulmonary pathophysiology are induced by two different strains of respiratory syncytial virus. Am. J. Pathol..

[53] Hancock G.E., Speelman D.J., Heers K., Bortell E., Smith J., Cosco C. (1996). Generation of atypical pulmonary inflammatory responses in BALB/c mice after immunization with the native attachment (G) glycoprotein of respiratory syncytial virus. J. Virol..

[54] Aung S., Tang Y.W., Graham B.S. (1999). Interleukin-4 diminishes CD8 (+) respiratory syncytial virus-specific cytotoxic T-lymphocyte activity in vivo. J. Virol..

[55] Schwarze J., Cieslewicz G., Joetham A., Ikemura T., Makela M.J., Dakhama A., Shultz L.D., Lamers M.C., Gelfand E.W. (2000). Critical roles for interleukin-4 and interleukin-5 during respiratory syncytial virus infection in the development of airway hyperresponsiveness after airway sensitization. Am. J. Respir. Crit. Care Med..

[56] Roman M., Calhoun W.J., Hinton K.L., Avendano L.F., Simon V., Escobar A.M., Gaggero A., Diaz P.V. (1997). Respiratory syncytial virus infection in infants is associated with predominant Th-2-like response. Am. J. Respir. Crit. Care Med..

[57] Legg J.P., Hussain I.R., Warner J.A., Johnston S.L., Warner J.O. (2003). Type 1 and type 2 cytokine imbalance in acute respiratory syncytial virus bronchiolitis. Am. J. Respir. Crit. Care Med..

[58] Chin J., Magoffin R.L., Shearer L.A., Schieble J.H., Lennette E.H. (1969). Field evaluation of a respiratory syncytial virus vaccine and a trivalent parainfluenza virus vaccine in a pediatric population. Am. J. Epidemiol..

[59] Kim H.W., Canchola J.G., Brandt C.D., Pyles G., Chanock R.M., Jensen K., Parrott R.H. (1969). Respiratory syncytial virus disease in infants despite prior administration of antigenic inactivated vaccine. Am. J. Epidemiol..

[60] Kapikian A.Z., Mitchell R.H., Chanock R.M., Shvedoff R.A., Stewart C.E. (1969). An epidemiologic study of altered clinical reactivity to respiratory syncytial (RS) virus infection in children previously vaccinated with an inactivated RS virus vaccine. Am. J. Epidemiol..

[61] Moghaddam A., Olszewska W., Wang B., Tregoning J.S., Helson R., Sattentau Q.J., Openshaw P.J. (2006). A potential molecular mechanism for hypersensitivity caused by formalin-inactivated vaccines. Nat. Med..

[62] Knudson C.J., Hartwig S.M., Meyerholz D.K., Varga S.M. (2015). RSV vaccine-enhanced disease is orchestrated by the combined actions of distinct CD4 T cell subsets. PLoS Pathog..

[63] Waris M.E., Tsou C., Erdman D.D., Zaki S.R., Anderson L.J. (1996). Respiratory synctial virus infection in BALB/c mice previously immunized with formalin-inactivated virus induces enhanced pulmonary inflammatory response with a predominant Th2-like cytokine pattern. J. Virol..

[64] Lee D.C., Harker J.A., Tregoning J.S., Atabani S.F., Johansson C., Schwarze J., Openshaw P.J. (2010). CD25+ natural regulatory T cells are critical in limiting innate and adaptive immunity and resolving disease following respiratory syncytial virus infection. J. Virol..

[65] Durant L.R., Makris S., Voorburg C.M., Loebbermann J., Johansson C., Openshaw P.J. (2013). Regulatory T cells prevent Th2 immune responses and pulmonary eosinophilia during respiratory syncytial virus infection in mice. J. Virol..

[66] Loebbermann J., Thornton H., Durant L., Sparwasser T., Webster K.E., Sprent J., Culley F.J., Johansson C., Openshaw P.J. (2012). Regulatory T cells expressing granzyme B play a critical role in controlling lung inflammation during acute viral infection. Mucosal Immunol..

[67] Loebbermann J., Durant L., Thornton H., Johansson C., Openshaw P.J. (2013). Defective immunoregulation in RSV vaccine-augmented viral lung disease restored by selective chemoattraction of regulatory T cells. Proc. Natl. Acad. Sci. USA.

[68] Akira S., Uematsu S., Takeuchi O. (2006). Pathogen recognition and innate immunity. Cell.

[69] Oh D.S., Kim T.H., Lee H.K. (2019). Differential Role of Anti-Viral Sensing Pathway for the Production of Type I Interferon beta in Dendritic Cells and Macrophages Against Respiratory Syncytial Virus A2 Strain Infection. Viruses.

[70] Rudd B.D., Schaller M.A., Smit J.J., Kunkel S.L., Neupane R., Kelley L., Berlin A.A., Lukacs N.W. (2007). MyD88-mediated instructive signals in dendritic cells regulate pulmonary immune responses during respiratory virus infection. J. Immunol..

[71] Kurt-Jones E.A., Popova L., Kwinn L., Haynes L.M., Jones L.P., Tripp R.A., Walsh E.E., Freeman M.W., Golenbock D.T., Anderson L.J. (2000). Pattern recognition receptors TLR4 and CD14 mediate response to respiratory syncytial virus. Nat. Immunol..

[72] Haynes L.M., Moore D.D., Kurt-Jones E.A., Finberg R.W., Anderson L.J., Tripp R.A. (2001). Involvement of toll-like receptor 4 in innate immunity to respiratory syncytial virus. J. Virol..

[73] Rallabhandi P., Phillips R.L., Boukhvalova M.S., Pletneva L.M., Shirey K.A., Gioannini T.L., Weiss J.P., Chow J.C., Hawkins L.D., Vogel S.N. (2012). Respiratory syncytial virus fusion protein-induced toll-like receptor 4 (TLR4) signaling is inhibited by the TLR4 antagonists Rhodobacter sphaeroides lipopolysaccharide and eritoran (E5564) and requires direct interaction with MD-2. MBio.

[74] Monick M.M., Yarovinsky T.O., Powers L.S., Butler N.S., Carter A.B., Gudmundsson G., Hunninghake G.W. (2003). Respiratory syncytial virus up-regulates TLR4 and sensitizes airway epithelial cells to endotoxin. J. Biol. Chem..

[75] Gagro A., Tominac M., Krsulovic-Hresic V., Bace A., Matic M., Drazenovic V., Mlinaric-Galinovic G., Kosor E., Gotovac K., Bolanca I. (2004). Increased Toll-like receptor 4 expression in infants with respiratory syncytial virus bronchiolitis. Clin. Exp. Immunol..

[76] Haeberle H.A., Takizawa R., Casola A., Brasier A.R., Dieterich H.J., van Rooijen N., Gatalica Z., Garofalo R.P. (2002). Respiratory syncytial virus-induced activation of nuclear factor-kappaB in the lung involves alveolar macrophages and toll-like receptor 4-dependent pathways. J. Infect. Dis..

[77] Tulic M.K., Hurrelbrink R.J., Prele C.M., Laing I.A., Upham J.W., le Souef P., Sly P.D., Holt P.G. (2007). TLR4 polymorphisms mediate impaired responses to respiratory syncytial virus and lipopolysaccharide. J. Immunol..

[78] Awomoyi A.A., Rallabhandi P., Pollin T.I., Lorenz E., Sztein M.B., Boukhvalova M.S., Hemming V.G., Blanco J.C., Vogel S.N. (2007). Association of TLR4 polymorphisms with symptomatic respiratory syncytial virus infection in high-risk infants and young children. J. Immunol..

[79] Murawski M.R., Bowen G.N., Cerny A.M., Anderson L.J., Haynes L.M., Tripp R.A., Kurt-Jones E.A., Finberg R.W. (2009). Respiratory syncytial virus activates innate immunity through Toll-like receptor 2. J. Virol..

[80] Arnold R., Konig W. (2006). Peroxisome proliferator-activated receptor-gamma agonists inhibit the replication of respiratory syncytial virus (RSV) in human lung epithelial cells. Virology.

[81] Kim T.H., Oh D.S., Jung H.E., Chang J., Lee H.K. (2019). Plasmacytoid Dendritic Cells Contribute to the Production of IFN-beta via TLR7-MyD88-Dependent Pathway and CTL Priming during Respiratory Syncytial Virus Infection. Viruses.

[82] Lukacs N.W., Smit J.J., Mukherjee S., Morris S.B., Nunez G., Lindell D.M. (2010). Respiratory virus-induced TLR7 activation controls IL-17-associated increased mucus via IL-23 regulation. J. Immunol..

[83] Groskreutz D.J., Monick M.M., Powers L.S., Yarovinsky T.O., Look D.C., Hunninghake G.W. (2006). Respiratory syncytial virus induces TLR3 protein and protein kinase R, leading to increased double-stranded RNA responsiveness in airway epithelial cells. J. Immunol..

[84] Rudd B.D., Smit J.J., Flavell R.A., Alexopoulou L., Schaller M.A., Gruber A., Berlin A.A., Lukacs N.W. (2006). Deletion of TLR3 alters the pulmonary immune environment and mucus production during respiratory syncytial virus infection. J. Immunol..

[85] Liu P., Jamaluddin M., Li K., Garofalo R.P., Casola A., Brasier A.R. (2007). Retinoic acid-inducible gene I mediates early antiviral response and Toll-like receptor 3 expression in respiratory syncytial virus-infected airway epithelial cells. J. Virol..

[86] Loo Y.M., Fornek J., Crochet N., Bajwa G., Perwitasari O., Martinez-Sobrido L., Akira S., Gill M.A., Garcia-Sastre A., Katze M.G. (2008). Distinct RIG-I and MDA5 signaling by RNA viruses in innate immunity. J. Virol..

[87] Demoor T., Petersen B.C., Morris S., Mukherjee S., Ptaschinski C., de Almeida Nagata D.E., Kawai T., Ito T., Akira S., Kunkel S.L. (2012). IPS-1 signaling has a nonredundant role in mediating antiviral responses and the clearance of respiratory syncytial virus. J. Immunol..

[88] Bhoj V.G., Sun Q., Bhoj E.J., Somers C., Chen X., Torres J.P., Mejias A., Gomez A.M., Jafri H., Ramilo O. (2008). MAVS and MyD88 are essential for innate immunity but not cytotoxic T lymphocyte response against respiratory syncytial virus. Proc. Natl. Acad. Sci. USA.

[89] Sabbah A., Chang T.H., Harnack R., Frohlich V., Tominaga K., Dube P.H., Xiang Y., Bose S. (2009). Activation of innate immune antiviral responses by Nod2. Nat. Immunol..

[90] Segovia J., Sabbah A., Mgbemena V., Tsai S.Y., Chang T.H., Berton M.T., Morris I.R., Allen I.C., Ting J.P., Bose S. (2012). TLR2/MyD88/NF-kappaB pathway, reactive oxygen species, potassium efflux activates NLRP3/ASC inflammasome during respiratory syncytial virus infection. PLoS ONE.

[91] Triantafilou K., Kar S., Vakakis E., Kotecha S., Triantafilou M. (2013). Human respiratory syncytial virus viroporin SH: A viral recognition pathway used by the host to signal inflammasome activation. Thorax.

[92] Goritzka M., Pereira C., Makris S., Durant L.R., Johansson C. (2015). T cell responses are elicited against Respiratory Syncytial Virus in the absence of signalling through TLRs, RLRs and IL-1R/IL-18R. Sci. Rep..

[93] Dalod M., Chelbi R., Malissen B., Lawrence T. (2014). Dendritic cell maturation: Functional specialization through signaling specificity and transcriptional programming. EMBO J..

[94] Johnson T.R., Johnson C.N., Corbett K.S., Edwards G.C., Graham B.S. (2011). Primary human mDC1, mDC2, and pDC dendritic cells are differentially infected and activated by respiratory syncytial virus. PLoS ONE.

[95] Guerrero-Plata A., Casola A., Suarez G., Yu X., Spetch L., Peeples M.E., Garofalo R.P. (2006). Differential response of dendritic cells to human metapneumovirus and respiratory syncytial virus. Am. J. Respir. Cell Mol. Biol..

[96] De Graaff P.M., de Jong E.C., van Capel T.M., van Dijk M.E., Roholl P.J., Boes J., Luytjes W., Kimpen J.L., van Bleek G.M. (2005). Respiratory syncytial virus infection of monocyte-derived dendritic cells decreases their capacity to activate CD4 T cells. J. Immunol..

[97] Jones A., Morton I., Hobson L., Evans G.S., Everard M.L. (2006). Differentiation and immune function of human dendritic cells following infection by respiratory syncytial virus. Clin. Exp. Immunol..

[98] Gupta M.R., Kolli D., Garofalo R.P. (2013). Differential response of BDCA-1+ and BDCA-3+ myeloid dendritic cells to respiratory syncytial virus infection. Respir. Res..

[99] Beyer M., Bartz H., Horner K., Doths S., Koerner-Rettberg C., Schwarze J. (2004). Sustained increases in numbers of pulmonary dendritic cells after respiratory syncytial virus infection. J. Allergy Clin. Immunol..

[100] Morris S., Swanson M.S., Lieberman A., Reed M., Yue Z., Lindell D.M., Lukacs N.W. (2011). Autophagy-mediated dendritic cell activation is essential for innate cytokine production and APC function with respiratory syncytial virus responses. J. Immunol..

[101] Lukens M.V., Kruijsen D., Coenjaerts F.E., Kimpen J.L., van Bleek G.M. (2009). Respiratory syncytial virus-induced activation and migration of respiratory dendritic cells and subsequent antigen presentation in the lung-draining lymph node. J. Virol..

[102] Plantinga M., Guilliams M., Vanheerswynghels M., Deswarte K., Branco-Madeira F., Toussaint W., Vanhoutte L., Neyt K., Killeen N., Malissen B. (2013). Conventional and monocyte-derived CD11b (+) dendritic cells initiate and maintain T helper 2 cell-mediated immunity to house dust mite allergen. Immunity.

[103] Lin K.L., Suzuki Y., Nakano H., Ramsburg E., Gunn M.D. (2008). CCR2+ monocyte-derived dendritic cells and exudate macrophages produce influenza-induced pulmonary immune pathology and mortality. J. Immunol..

[104] Neyt K., Lambrecht B.N. (2013). The role of lung dendritic cell subsets in immunity to respiratory viruses. Immunol. Rev..

[105] Guilliams M., Lambrecht B.N., Hammad H. (2013). Division of labor between lung dendritic cells and macrophages in the defense against pulmonary infections. Mucosal Immunol..

[106] Minoda Y., Virshup I., Leal Rojas I., Haigh O., Wong Y., Miles J.J., Wells C.A., Radford K.J. (2017). Human CD141 (+) Dendritic Cell and CD1c (+) Dendritic Cell Undergo Concordant Early Genetic Programming after Activation in Humanized Mice In Vivo. Front. Immunol..

[107] Edelson B.T., Wumesh K.C., Juang R., Kohyama M., Benoit L.A., Klekotka P.A., Moon C., Albring J.C., Ise W., Michael D.G. (2010). Peripheral CD103+ dendritic cells form a unified subset developmentally related to CD8alpha+ conventional dendritic cells. J. Exp. Med..

[108] Hochrein H., Shortman K., Vremec D., Scott B., Hertzog P., O’Keeffe M. (2001). Differential production of IL-12, IFN-alpha, and IFN-gamma by mouse dendritic cell subsets. J. Immunol..

[109] Maldonado-Lopez R., de Smedt T., Michel P., Godfroid J., Pajak B., Heirman C., Thielemans K., Leo O., Urbain J., Moser M. (1999). CD8alpha+ and CD8alpha- subclasses of dendritic cells direct the development of distinct T helper cells in vivo. J. Exp. Med..

[110] Martinez-Lopez M., Iborra S., Conde-Garrosa R., Sancho D. (2015). Batf3-dependent CD103+ dendritic cells are major producers of IL-12 that drive local Th1 immunity against Leishmania major infection in mice. Eur. J. Immunol..

[111] Igyarto B.Z., Haley K., Ortner D., Bobr A., Gerami-Nejad M., Edelson B.T., Zurawski S.M., Malissen B., Zurawski G., Berman J. (2011). Skin-resident murine dendritic cell subsets promote distinct and opposing antigen-specific T helper cell responses. Immunity.

[112] Durai V., Murphy K.M. (2016). Functions of Murine Dendritic Cells. Immunity.

[113] Hildner K., Edelson B.T., Purtha W.E., Diamond M., Matsushita H., Kohyama M., Calderon B., Schraml B.U., Unanue E.R., Diamond M.S. (2008). Batf3 deficiency reveals a critical role for CD8alpha+ dendritic cells in cytotoxic T cell immunity. Science.

[114] Ruckwardt T.J., Morabito K.M., Bar-Haim E., Nair D., Graham B.S. (2018). Neonatal mice possess two phenotypically and functionally distinct lung-migratory CD103 (+) dendritic cell populations following respiratory infection. Mucosal Immunol..

[115] Ruckwardt T.J., Malloy A.M., Morabito K.M., Graham B.S. (2014). Quantitative and qualitative deficits in neonatal lung-migratory dendritic cells impact the generation of the CD8+ T cell response. PLoS Pathog..

[116] Ruckwardt T.J., Malloy A.M., Gostick E., Price D.A., Dash P., McClaren J.L., Thomas P.G., Graham B.S. (2011). Neonatal CD8 T-cell hierarchy is distinct from adults and is influenced by intrinsic T cell properties in respiratory syncytial virus infected mice. PLoS Pathog..

[117] Thornburg N.J., Shepherd B., Crowe J.E. (2010). Transforming growth factor beta is a major regulator of human neonatal immune responses following respiratory syncytial virus infection. J. Virol..

[118] Dudziak D., Kamphorst A.O., Heidkamp G.F., Buchholz V.R., Trumpfheller C., Yamazaki S., Cheong C., Liu K., Lee H.W., Park C.G. (2007). Differential antigen processing by dendritic cell subsets in vivo. Science.

[119] Beaty S.R., Rose C.E., Sung S.S. (2007). Diverse and potent chemokine production by lung CD11bhigh dendritic cells in homeostasis and in allergic lung inflammation. J. Immunol..

[120] Briseno C.G., Satpathy A.T., Davidson J.T.t., Ferris S.T., Durai V., Bagadia P., O’Connor K.W., Theisen D.J., Murphy T.L., Murphy K.M. (2018). Notch2-dependent DC2s mediate splenic germinal center responses. Proc. Natl. Acad. Sci. USA.

[121] Lewis K.L., Caton M.L., Bogunovic M., Greter M., Grajkowska L.T., Ng D., Klinakis A., Charo I.F., Jung S., Gommerman J.L. (2011). Notch2 receptor signaling controls functional differentiation of dendritic cells in the spleen and intestine. Immunity.

[122] Kallal L.E., Schaller M.A., Lindell D.M., Lira S.A., Lukacs N.W. (2010). CCL20/CCR6 blockade enhances immunity to RSV by impairing recruitment of DC. Eur. J. Immunol..

[123] Shrestha B., You D., Saravia J., Siefker D.T., Jaligama S., Lee G.I., Sallam A.A., Harding J.N., Cormier S.A. (2017). IL-4Ralpha on dendritic cells in neonates and Th2 immunopathology in respiratory syncytial virus infection. J. Leukoc. Biol..

[124] Ripple M.J., You D., Honnegowda S., Giaimo J.D., Sewell A.B., Becnel D.M., Cormier S.A. (2010). Immunomodulation with IL-4R alpha antisense oligonucleotide prevents respiratory syncytial virus-mediated pulmonary disease. J. Immunol..

[125] Wang H., Peters N., Schwarze J. (2006). Plasmacytoid dendritic cells limit viral replication, pulmonary inflammation, and airway hyperresponsiveness in respiratory syncytial virus infection. J. Immunol..

[126] Schijf M.A., Lukens M.V., Kruijsen D., van Uden N.O., Garssen J., Coenjaerts F.E., van’t Land B., van Bleek G.M. (2013). Respiratory syncytial virus induced type I IFN production by pDC is regulated by RSV-infected airway epithelial cells, RSV-exposed monocytes and virus specific antibodies. PLoS ONE.

[127] Smit J.J., Lindell D.M., Boon L., Kool M., Lambrecht B.N., Lukacs N.W. (2008). The balance between plasmacytoid DC versus conventional DC determines pulmonary immunity to virus infections. PLoS ONE.

[128] Smit J.J., Rudd B.D., Lukacs N.W. (2006). Plasmacytoid dendritic cells inhibit pulmonary immunopathology and promote clearance of respiratory syncytial virus. J. Exp. Med..

[129] Cormier S.A., Shrestha B., Saravia J., Lee G.I., Shen L., DeVincenzo J.P., Kim Y.I., You D. (2014). Limited type I interferons and plasmacytoid dendritic cells during neonatal respiratory syncytial virus infection permit immunopathogenesis upon reinfection. J. Virol..

[130] Lynch J.P., Werder R.B., Loh Z., Sikder M.A.A., Curren B., Zhang V., Rogers M.J., Lane K., Simpson J., Mazzone S.B. (2018). Plasmacytoid dendritic cells protect from viral bronchiolitis and asthma through semaphorin 4a-mediated T reg expansion. J. Exp. Med..

[131] Hobson L., Everard M.L. (2008). Persistent of respiratory syncytial virus in human dendritic cells and influence of nitric oxide. Clin. Exp. Immunol..

[132] Hendricks D.A., Baradaran K., McIntosh K., Patterson J.L. (1987). Appearance of a soluble form of the G protein of respiratory syncytial virus in fluids of infected cells. J. Gen. Virol..

[133] Chirkova T., Boyoglu-Barnum S., Gaston K.A., Malik F.M., Trau S.P., Oomens A.G., Anderson L.J. (2013). Respiratory syncytial virus G protein CX3C motif impairs human airway epithelial and immune cell responses. J. Virol..

[134] Spann K.M., Tran K.C., Collins P.L. (2005). Effects of nonstructural proteins NS1 and NS2 of human respiratory syncytial virus on interferon regulatory factor 3, NF-kappaB, and proinflammatory cytokines. J. Virol..

[135] Teng M.N., Whitehead S.S., Bermingham A., St Claire M., Elkins W.R., Murphy B.R., Collins P.L. (2000). Recombinant respiratory syncytial virus that does not express the NS1 or M2-2 protein is highly attenuated and immunogenic in chimpanzees. J. Virol..

[136] Boyapalle S., Wong T., Garay J., Teng M., San Juan-Vergara H., Mohapatra S., Mohapatra S. (2012). Respiratory syncytial virus NS1 protein colocalizes with mitochondrial antiviral signaling protein MAVS following infection. PLoS ONE.

[137] Ling Z., Tran K.C., Teng M.N. (2009). Human respiratory syncytial virus nonstructural protein NS2 antagonizes the activation of beta interferon transcription by interacting with RIG-I. J. Virol..

[138] Bossert B., Marozin S., Conzelmann K.K. (2003). Nonstructural proteins NS1 and NS2 of bovine respiratory syncytial virus block activation of interferon regulatory factor 3. J. Virol..

[139] Ren J., Liu T., Pang L., Li K., Garofalo R.P., Casola A., Bao X. (2011). A novel mechanism for the inhibition of interferon regulatory factor-3-dependent gene expression by human respiratory syncytial virus NS1 protein. J. Gen. Virol..

[140] Lo M.S., Brazas R.M., Holtzman M.J. (2005). Respiratory syncytial virus nonstructural proteins NS1 and NS2 mediate inhibition of Stat2 expression and alpha/beta interferon responsiveness. J. Virol..

[141] Elliott J., Lynch O.T., Suessmuth Y., Qian P., Boyd C.R., Burrows J.F., Buick R., Stevenson N.J., Touzelet O., Gadina M. (2007). Respiratory syncytial virus NS1 protein degrades STAT2 by using the Elongin-Cullin E3 ligase. J. Virol..

[142] Ramaswamy M., Shi L., Varga S.M., Barik S., Behlke M.A., Look D.C. (2006). Respiratory syncytial virus nonstructural protein 2 specifically inhibits type I interferon signal transduction. Virology.

[143] Whelan J.N., Tran K.C., van Rossum D.B., Teng M.N. (2016). Identification of Respiratory Syncytial Virus Nonstructural Protein 2 Residues Essential for Exploitation of the Host Ubiquitin System and Inhibition of Innate Immune Responses. J. Virol..

[144] Munir S., le Nouen C., Luongo C., Buchholz U.J., Collins P.L., Bukreyev A. (2008). Nonstructural proteins 1 and 2 of respiratory syncytial virus suppress maturation of human dendritic cells. J. Virol..

[145] Munir S., Hillyer P., le Nouen C., Buchholz U.J., Rabin R.L., Collins P.L., Bukreyev A. (2011). Respiratory syncytial virus interferon antagonist NS1 protein suppresses and skews the human T lymphocyte response. PLoS Pathog..

[146] Gonzalez P.A., Prado C.E., Leiva E.D., Carreno L.J., Bueno S.M., Riedel C.A., Kalergis A.M. (2008). Respiratory syncytial virus impairs T cell activation by preventing synapse assembly with dendritic cells. Proc. Natl. Acad. Sci. USA.

[147] Cespedes P.F., Bueno S.M., Ramirez B.A., Gomez R.S., Riquelme S.A., Palavecino C.E., Mackern-Oberti J.P., Mora J.E., Depoil D., Sacristan C. (2014). Surface expression of the hRSV nucleoprotein impairs immunological synapse formation with T cells. Proc. Natl. Acad. Sci. USA.

[148] Thornburg N.J., Hayward S.L., Crowe J.E. (2012). Respiratory syncytial virus regulates human microRNAs by using mechanisms involving beta interferon and NF-kappaB. MBio.

[149] Bakre A., Mitchell P., Coleman J.K., Jones L.P., Saavedra G., Teng M., Tompkins S.M., Tripp R.A. (2012). Respiratory syncytial virus modifies microRNAs regulating host genes that affect virus replication. J. Gen. Virol..

[150] Malloy A.M., Ruckwardt T.J., Morabito K.M., Lau-Kilby A.W., Graham B.S. (2017). Pulmonary Dendritic Cell Subsets Shape the Respiratory Syncytial Virus-Specific CD8+ T Cell Immunodominance Hierarchy in Neonates. J. Immunol..

[151] McLellan J.S., Chen M., Joyce M.G., Sastry M., Stewart-Jones G.B., Yang Y., Zhang B., Chen L., Srivatsan S., Zheng A. (2013). Structure-based design of a fusion glycoprotein vaccine for respiratory syncytial virus. Science.

[152] Crank M.C., Ruckwardt T.J., Chen M., Morabito K.M., Phung E., Costner P.J., Holman L.A., Hickman S.P., Berkowitz N.M., Gordon I.J. (2019). A proof of concept for structure-based vaccine design targeting RSV in humans. Science.

[153] Marcandalli J., Fiala B., Ols S., Perotti M., de van der Schueren W., Snijder J., Hodge E., Benhaim M., Ravichandran R., Carter L. (2019). Induction of Potent Neutralizing Antibody Responses by a Designed Protein Nanoparticle Vaccine for Respiratory Syncytial Virus. Cell.

